# Optimization and Standardization of the Extraction Method of *Balanites aegyptiaca* Del. Seeds (Zygophyllaceae) Used in the Formulation of an Antiparasitic Phytomedicine

**DOI:** 10.3390/ph17121698

**Published:** 2024-12-17

**Authors:** Mohamed Bonewendé Belemlilga, Salfo Ouedraogo, Gilchrist Abdoul Laurent Boly, Do Harouna Dao, Jonas Tiami Coulibaly, Jean Claude Romaric Pingdwindé Ouedraogo, Souleymane Compaoré, Sidiki Traore, Moumouni Koala, Estelle Noëla Hoho Youl, Lazare Belemnaba, Félix Bondo Kini, Aristide Traore, Séni Kouanda, Sylvin Ouedraogo

**Affiliations:** 1 Laboratoire de Recherche-Développement de Phytomédicaments et Médicaments (LR-D/PM), Institut de Recherche en Sciences de la Santé (IRSS), Centre National de la Recherche Scientifique et Technologique (CNRST), Ouagadougou 03 BP 7047, Burkina Faso; ouedraogosalfo35@yahoo.fr (S.O.); belemnabalaz@yahoo.fr (L.B.);; 2 Laboratoire de Développement du Médicament (LADME), Centre de Formation, de Recherche et d’Expertises en Sciences du Médicament (CEA-CFOREM), École Doctorale Sciences de la Santé (ED2S), Université Joseph KI-ZERBO, Ouagadougou 03 BP 7021, Burkina Faso; 3 Département Biomédical et Santé Publique, Institut de Recherche en Sciences de la Santé (IRSS), Centre National de la Recherche Scientifique et Technologique (CNRST), Ouagadougou 03 BP 7047, Burkina Faso; senikouanda@gmail.com

**Keywords:** optimization, standardization, *Balanites aegyptiaca*, antiparasitic phytomedicine

## Abstract

**Background/Objectives:** *Balanites aegyptiaca* Del. (Zygophyllaceae) is widely used in traditional medicine, both human and veterinary, throughout Africa for its many properties, including antiparasitic properties. This experimental study aims to optimize the extraction conditions of the seeds of *Balanites aegyptiaca* Del. **Methods:** Aqueous maceration was carried out with mass-to-volume ratios of 40%, 30%, 20%, 10% and 5% and extraction times of 6, 12, 24, 36 and 48 h. Extraction yields, phytochemical screening, saponins assay, antioxidant activities ABTS+ free radical scavenging activities, Ferric-reducing antioxidant power (FRAP) assay and antiparasitic tests on *Heligmosomoides bakeri* were used to compare the different extracts. **Results:** The pharmaco-chemical study generally showed that aqueous maceration gave the best results, with a mass/volume ratio of 10% after 12 h of maceration. The yield obtained was 28.03% with a saponins content of 13.81 mg/g. The antioxidant activities were 4.25 ± 0.17 µg/mL by the ABTS method and 0.739 µg/mL by the FRAP method. The larvicidal activity also showed that the 10% 12 h extract produced 100% larval mortality from 25 µg/mL. **Conclusions:** These data provide a basis for guiding the extraction process parameters in producing this antiparasitic phytomedicine.

## 1. Introduction

According to the World Health Organization (WHO), in some developing countries in Asia, Africa and Latin America, 80% of the population relies on traditional medicine, especially in rural areas, because of the proximity and accessibility of this type of care at an affordable cost and mainly because of the lack of access to modern medicine for these populations [1]. This situation is based on traditional medical knowledge deeply rooted in the local culture. The development of this traditional medical knowledge offers a wealth of potential and prospects in healthcare for African countries [2,3].

With scientific progress, phytotherapy is evolving toward modern phytotherapy, also known as “rational phytotherapy” or “medical phytotherapy”, which uses modern methods to extract the active ingredients contained in medicinal plants and validates their beneficial properties for health through a scientific approach of biochemical and pharmacological analyses supported by computer power, as well as clinical trials [4,5,6,7,8].

Modern herbal medicine is based on scientific evidence and uses active plant extracts, standardized and marketed as finished products in phytomedicines. Phytomedicines (PMs) and improved traditional medicines (ITMs) are a vital alternative to health spending in most African countries, which are still 90% dependent on foreign pharmaceutical companies and laboratories [9].

Because of the high medical, scientific and socio-economic stakes involved in the industrial exploitation of research results, the “Institut de Recherche en Sciences de la Santé (IRSS)” has developed a phytomedicine based on the seeds of *Balanites aegyptiaca* (*B. aegyptiaca*). *B. aegyptiaca*, or desert date palm, is a very thorny phanerophyte found in the arid regions of tropical Africa, from the Sahara to Palestine, Arabia and India. *Balanites aegyptiaca* is widely used in traditional human and veterinary medicine throughout Africa for its many properties [10]. Throughout the world, the different parts of this plant are used to treat many diseases, such as bacterial and fungal infections, hemorrhagic menstruation, goiter, bilharzia, malaria, colic, yellow fever, hematuria, hydrocele, hemorrhoids, dermatitis, abdominal pain, colds, diabetes, arterial hypertension and helminthic infections [11,12,13,14,15,16,17]. Numerous studies based on ethnopharmacology have reported evidence of using *B. aegyptiaca* seeds in treating parasitosis for over twenty years [17,18,19,20,21,22,23,24]. Several studies have also been carried out at the IRSS on extracts of the plant, demonstrating the antiparasitic properties of its seeds [25,26,27,28]. Based on the results of his study, Gnoula et al. hypothesized that the main nematocidal agent present in the seeds of *B. aegyptiaca* may be balanitin-7 (Bal-7), a heteroside of the diosgenyl saponoside family [27]. However, the method of obtaining these extracts still needs to be optimized and standardized in order to obtain products that meet quality standards, have higher yields and are safe for industrial production. The aim of this work is to optimize the conditions for obtaining freeze-dried *B. aegyptiaca* seeds for the formulation of phytomedicines by studying several factors that may affect the yield and quality of the final extract.

## 2. Results

### 2.1. Figures and Tables

#### 2.1.1. Phytochemical Studies

**Figure 1:**
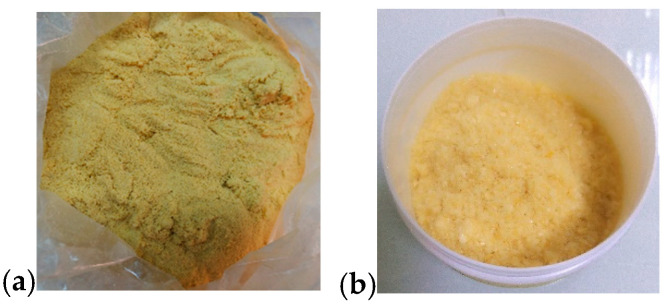
Almond powder (**a**) and lyophilized extract (**b**) of *Balanites aegyptiaca* seeds.

The residual moisture content of lyophilizate, determined by drying at 105 °C for 15 min, is shown in Figure 1.

The macroscopic and organoleptic characteristics of the plant material and freeze-dried extracts are shown in Table 1.

**Figure 2:**
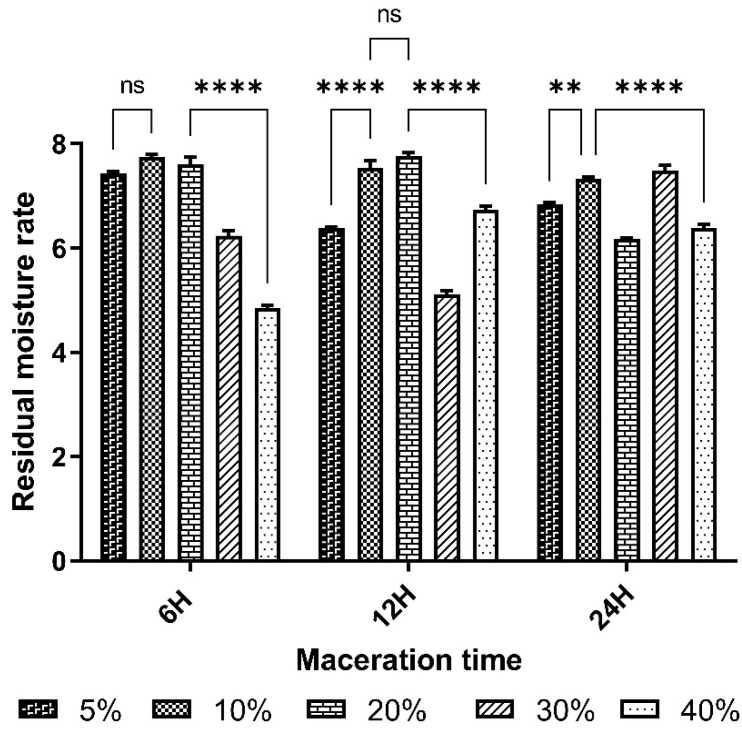
The residual moisture content of different lyophilizate. ** *p* < 0.01, **** *p* < 0.0001 is considered significant compared with the other % macerates (two-way ANOVA followed by the “Tukey” multiple comparison test; ns is considered not significant). *n* = 3.

The residual moisture content of lyophilized extracts determined by a halogen desiccator (RADWAG) at 105 °C for 15 min is shown in Figure 2.

The residual moisture content of the plant material was 4.84 ± 0.06. Results for the residual moisture content of lyophilized extracts showed values below 10%. These values ranged from 5.11 ± 0.10 to 7.76 ± 0.08%. The RMC of lyophilized extracts is relatively higher than that of the plant material.

The pH values of the powders are given in Table 2.

The plant material had a pH of 6.99 ± 0.02, and the pH of the extracts ranged from 6.43 to 6.99.

**Figure 3:**
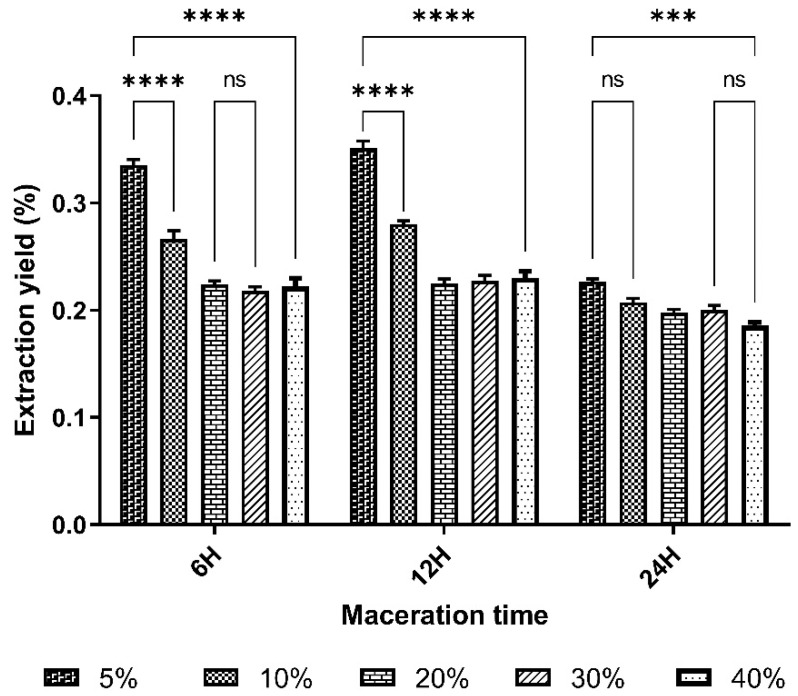
Extraction yield histogram of aqueous macerates mass/volume ratio as a function of time (hours). *** *p* < 0.01, **** *p* < 0.0001 is considered significant compared with the other % macerates (two-way ANOVA followed by the “Tukey” multiple comparison test; ns is considered not significant). *n* = 3.

Results of extraction yield are expressed as a percentage (%) and are shown in Table 3 and Figure 3 below.

The macerations carried out at 36 and 48 h were unsuccessful due to contamination. In fact, during handling, the filtrates obtained with macerations at 36 and 48 h had slimy aspects and odors characteristic of putrefaction. Maceration yields ranged from 18.56 ± 1.2% to 35.12 ± 1.1%. The highest yield (35.12 ± 1.1%) was obtained with a mass-to-volume ratio of 10% at 12 h.

The yields of the 5% 6 h and 10% 12 h mass/volume ratios differed only slightly from those of the 10% 6 h and 5% 12 h mass/volume ratios and very significantly from the other yields.

There was no significant difference between the yields obtained with the 20%, 30% and 40% mass/volume ratios.

**Figure 4:**
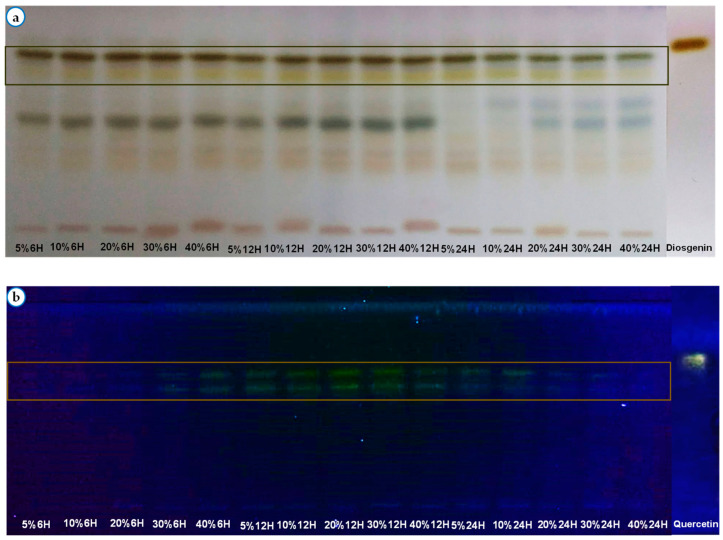
Analytical TLC profile of different extracts. (**a**) The presence of saponins revealed by sulfuric anisaldehyde after heating the plate observed in visible light. (**b**) Presence of flavonoids (at 254 nm) detected by NEU reagent.

The results of phytochemical screening in tubes and by TLC are shown in Table 4 and Table 5 and Figure 4.

Secondary metabolites such as saponins and flavonoids were detected in lyophilized extracts. All extracts had the same chromatographic profiles but with spots of different intensity, as shown in the chromatographic profile of flavonoids and saponins.

The saponins content of the extracts ranged from 9.27 ± 0.07 mg/g to 13.81 ± 0.04 mg/g. Analyses show that the highest contents were obtained with m/v ratios of 10% and 5% at 12 o’clock: 13.81 ± 0.04 mg/g and 12.32 ± 0.07 mg/g, respectively.

Analysis of all these results shows that the 10% m/v ratio at 12 h had the highest saponins content (13.81 ± 0.04 mg/g).

#### 2.1.2. Pharmacological Studies

The concentrations of the extracts measured by the ABTS method are shown in Table 6. The 50% inhibitory concentration (IC_50_) of Trolox was 2.63 ± 0.04. The results of the ferric-reducing antioxidant power (FRAP) assay are shown in Table 7.

The ABTS radical reduction method gave an IC_50_ for lyophilized extracts ranging from 3.57 ± 0.21 mg/mL to 4.82 ± 0.10 mg/mL at the 5 mg/mL extract concentration and from 2.63 ± 0.04 ug/mL at the 1 mg/mL concentration for the reference trolox. The 20% and 30% 24 h extracts have the lowest IC_50_ of 3.57 ± 0.21 mg/mL and 3.60 ± 0.20 mg/mL, respectively.

There was no significant difference in the reducing power of the different extracts.

**Figure 5:**
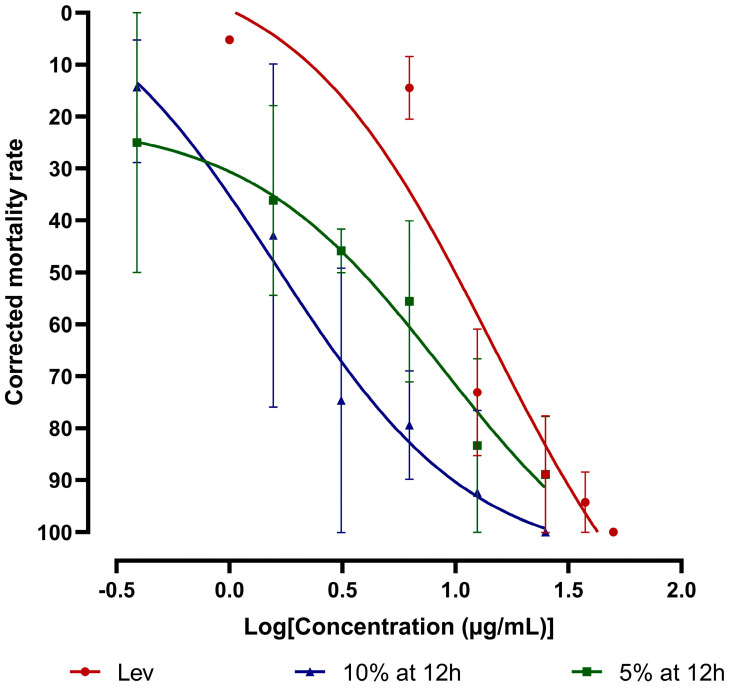
Larvicidal effect of *B. aegyptiaca* extracts and the standard on *H. bakeri*.

The larvicidal activity of the best optimized extracts on *H. bakeri* is shown in Figure 5.

The 50% inhibitory concentrations of extracts and standards are shown below:

IC_50_ = 14.52 µg/mL for Lev, 8.805 µg/mL for 5% extract and 1.605 µg/mL for 10% extract.

## 3. Discussion

Our work has enabled us to obtain relevant results. However, to improve the compounds’ phytochemical profile, it is important to carry out an HPLC analysis of the various freeze-dried extracts compared with reference molecules already isolated from plants.

The plant material was yellow, with a coarse texture, a very characteristic odor and a bitter taste (Figure 1). Aqueous extraction was performed as recommended by the health traditional practitioner. Water, the most polar solvent, was used to extract a wide range of polar compounds. It has the advantage of dissolving many substances; it is inexpensive, non-toxic, non-flammable and highly polar [29,30]. The lyophilizates varied in color from yellow to light yellow, depending on the mass-to-volume ratio, with a bitter-sweet taste, the sweetness being more pronounced in lyophilizates with a low mass-to-volume ratio and a fine texture. These changes in characteristics are thought to be related to extraction [31]. Organoleptic and macroscopic characteristics are parameters used in raw materials’ identification and quality control (Table 1). These data help to establish quality control and assurance standards and to define the purity of herbal or synthetic drugs [32]. Visual assessment of appearance sometimes allows rapid identification of certain herbal drugs, checking their degree of purity according to the presence or absence of foreign elements, molds, etc., and possibly detecting adulteration or falsification. A color change may indicate deterioration due to poor drying or storage conditions [30,33].

The result for the residual moisture content of the plant material was 4.84 ± 0.06 (Figure 2). The plant material had a pH of 6.99 ± 0.02, and the pH of the extracts ranged from 6.43 to 6.99. These values were relatively stable and did not significantly vary (Table 2). The control of this parameter reduces errors in estimating the actual weight of the plant material and guarantees the quality during the storage period [34]. This value, below 10%, indicates that the powder is sufficiently dry and can be stored during the handling period without the development of molds or yeasts, according to the standards of the European Pharmacopoeia [32]. In fact, too high a water content (above 10%) can promote enzymatic reactions with negative consequences on the appearance of the plant drug, its organoleptic characteristics and its therapeutic properties during the shelf life of the plant material powder. High residual moisture also favors the proliferation of microorganisms such as bacteria, yeasts and molds. These results are similar to those obtained by Sanfo [25], who obtained RMC below 10%, i.e., 4.26%. The RMC of lyophilizates varied from 5.11% to 7.76%, with an average of 6.65%. These lyophilizates were more or less dry and could be stored without mold or yeast growth. The best yields, 35.12 ± 1.1% and 33.54%, respectively, were obtained from extracts macerated for 12 h and 6 h at a mass/volume ratio of 5% (Table 3 and Figure 3).

The mass/volume ratio parameter has a significant effect on yield. The yield results show that increasing the solvent volume by decreasing the m/v ratio improves the extraction yield up to a ratio of 5%; in fact, the best yields are obtained with the lowest m/v ratio. This is consistent with the principle of mass transfer, which states that the driving force during extraction is the concentration gradient between the solid and the external liquid medium. This force becomes important when the liquid/solid ratio used is higher [35]. Similar results were obtained by Cacace who evaluated the effect of the solids ratio on the extraction rates in their studies [35]. They found that increasing the volume of the solvent had a positive effect on extraction regardless of the type of the solvent used.

Increasing the extraction time can often improve the extraction yield. This is mainly due to the fact that a longer contact time between the sample and the solvent allows a better solubilization of the target compounds in the solvent. This more efficient solubilization leads to a more complete extraction of the desired compounds, thus increasing the overall extraction yield [36]. We can state that a long extraction time would allow for good extraction and therefore good yield.

However, in this study, after 6 h, increasing the time did not significantly improve the yield. This can be explained by the phenomenon of extraction saturation when time is extended over a long period [37]. This result is similar to that of Tiendrebeogo who found that the time parameter alone in maceration was not sufficient to significantly improve extraction [37]. The results of our study show that the condition for optimal crude yield from the aqueous maceration of *B. aegyptiaca* seeds crush would be maceration for 6 h at a mass/volume ratio of 5%. According to Furlan and Bren, the choice of isolation and extraction method significantly affects the composition of the obtained extract [38].

Phytochemical screening of the extracts by thin-layer chromatography on chromatographic plate using the methods described by Wagner and Bladt revealed metabolites such as saponins and flavonoids. Saponins were the most essential compounds in the extracts, as significant precipitation reactions were obtained with appropriate reagents. Chemical screening revealed spots of equal intensity in all extracts (Table 4 and Figure 4). Assay results ranged from 9.27 ± 0.07 mg/g to 13.81 ± 0.04 mg/g. Analyses show that the highest levels were obtained at the 12 h maceration time with 13.81 ± 0.04 mg/g at 10%, 12.89 ± 0.06 mg/g at 30%, 12.59 ± 0.02 mg/g at 40% and 12.32 ± 0.07 mg/g at 5% (Table 5). The most significant content obtained with 12 h time parameters and a 10% mass/volume ratio was 13.81 ± 0.04 mg/g. The authors have already highlighted the phytochemical groups found in the plant’s seeds [25,27,39].

The antioxidant activity results by ABTS ranged from 3.57 ± 0.21 μg/mL to 4.82 ± 0.10 μg/mL. The most active extracts (4.82 ± 0.10), (4.54 ± 0.13) and (4.52 ± 0.19) were obtained at 6 h of the 5% and 10% ratios and 12 h of the 5% ratio, respectively (Table 6 and Table 7). The lower the concentration (IC_50_), the higher the antioxidant effect [40]. The phytochemical groups identified, namely flavonoids, tannins and saponins, are thought to be responsible for the antioxidant activity of the extracts [41,42].

These tests allowed us to determine the anthelmintic activity of the two *B. aegyptiaca* seeds extracts with the highest yields while maintaining their physicochemical properties against *H. bakeri* L_1_ larvae [43,44]. The results obtained show an interesting larvicidal effect. Indeed, the 5% extract showed a maximum effect of 84.64% larval mortality at a concentration of 25 µg/mL, while the 10% extract resulted in 100% mortality of *H. bakeri* L_1_ larvae at the same concentration—also, IC_50_ (extract at 5%) < IC_50_ (extract at 10%) (Figure 5). Therefore, we can say that the 10% extract at 12 h is more effective and potent than the 5% extract. The difference in the activity of the extracts tested may be due to the content of secondary metabolites, especially saponins, which are antiparasitic substances present in the extracts [45,46].

*B. aegyptiaca* has been chemically investigated for various classes of constituents. It is reported to contain several secondary metabolites and bioactive compounds, including flavonoids, alkaloids, glucosides, phenols, steroids, saponins, furanocoumarins, diosgenin, N-trans-feruloyl tyramine, N-cis-feruloyl tyramine, trigonelline, balanitol and fatty acid [27,47]. The 10% extract at 12 h has the highest saponins content compared with the other extracts. In *B. aegyptiaca* fruits, saponins such as balanins 4, 5, 6 and 7 have been isolated.

The activity of the different extracts obtained by successive depletion of *B. aegyptiaca* seeds powder was evaluated on *C. elegans*. Aqueous and methanolic extracts resulted in worm mortality. However, the aqueous extract (IC_50_ = 1.0 mg/mL) was much more nematocidal on *C. elegans* than the methanolic extract (IC_50_ = 25.3 mg/mL). Pure balanitin-7 obtained after successive column fractions showed very high nematocidal activity (IC_50_ = 0.1 μg/mL, expressed as aqueous extract equivalent) [27]. Based on the results of his study, Gnoula hypothesized that the significant nematocidal agent present in the seeds of *B. aegyptiaca* may be balanitin-7 (Bal-7), a heteroside of the diosgenin saponins family [27]. Since the 10% 12 h extract is rich in saponins, we can assume that the larvicidal activity on *H. bakeri* is related to the high presence of this phytochemical group. Saponins possess bioactivities that make them valuable in the pharmaceutical industry as antiparasitic, antimicrobial, antiviral and anti-inflammatory agents [48,49].

## 4. Materials and Methods

### 4.1. Plant Materials

**Figure 6:**
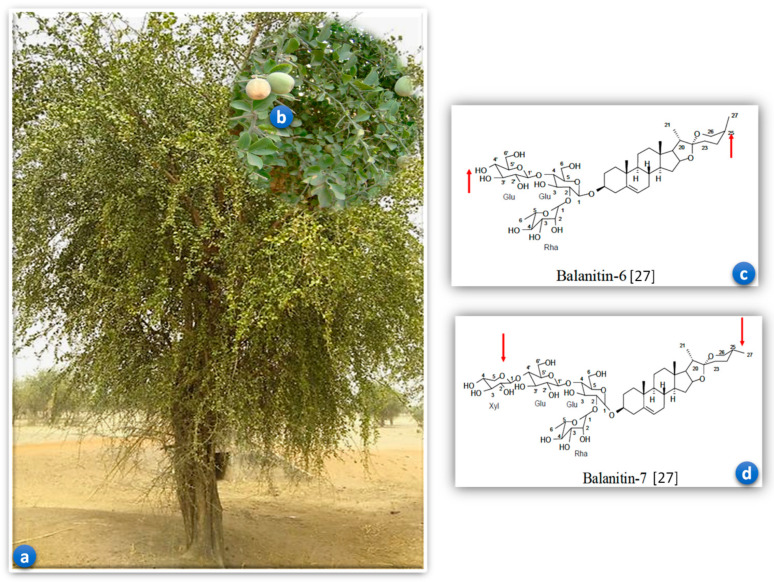
Whole plant (**a**), fruits (**b**) and isolated saponins (**c**,**d**) of *Balanites aegyptiaca*.

The plant’s raw material is the fruit of *B. aegyptiaca*. These fruits were harvested in March 2021 in Manga, in the south-central region of Burkina Faso, 100 km from the capital Ouagadougou. A plant sample was collected and identified at the Joseph KI-ZERBO University Herbarium under identification number 17928 with herbarium reference number 6916. Quality control was performed on *B. aegyptiaca* seed vegetal drug and lyophilizate. Figure 6 shows the whole plant, fruits and isolated saponins of *B. aegyptiaca* [27].

### 4.2. Quality Control of Vegetal Drug

*Macroscopic and organoleptic characteristics*: The organoleptic characteristics (taste and smell) were determined by tasting and sniffing the powder [50].

Determination of pH: The pH was determined by putting the pH-meter electrode (Eutech, Singapore) in 1% (*w*/*v*) aqueous solutions of each vegetable material (thrice). The test was performed in triplicate, and the mean and standard deviation were calculated (m ± standard deviation, *n* = 3).

*Residual moisture content (RMC)*: The residual moisture content of the powders was determined according to the thermogravimetric method of the European Pharmacopoeia 10th edition in an oven [32]. The assay was performed in triplicate on one (01) gram of powder. The mean and standard deviation were calculated (*n* = 3, mean, standard deviation).

*Total ash content*: Total ash levels were determined according to the European Pharmacopoeia 10th edition by calcining one (01) gram of each plant powder in a furnace (Bouvier, Belgium) at about 600 °C. The total ash content was expressed as percentage.

*Microbiological quality*: The microbial loads assayed were total microbial flora, Salmonella and thermo-tolerant coliforms. Total microbial flora and Salmonella were determined by the method of the European Pharmacopoeia 6th edition. Thermo-tolerant coliforms were determined according to ISO 7218. Colony counts were performed for calculations of the number of colony-forming units per gram (CFU/g).

### 4.3. Quality Control of Lyophilizate

It involves the maceration of *B. aegyptiaca* almond powder, varying the parameters of the mass/volume ratio and maceration time to obtain a higher yield of lyophilization, using a technique that does not involve the use of an organic solvent, such as cryoconcentration. A 50 g test sample of *B. aegyptiaca* seed powder is macerated in different volumes of distilled water (1000 mL, 500 mL, 250 mL, 165 mL and 125 mL) at ratios of 5%, 10%, 20%, 30% and 40% and manually stirred. The mixture is macerated at room temperature for 6, 12, 24 or 48 h.

The extracts obtained are filtered through a nylon filter at the end of the maceration time. The filtrates are delipidated by freezing for 5 h, and then the supernatant (lipid) is removed. The filtrates are centrifuged at 2000 rpm for 10 min and filtered again. The final filtrates are frozen and lyophilized for further analysis.

#### 4.3.1. Macroscopic and Organoleptic Characteristics

Characteristics such as texture, color, taste and odor were determined by the method described in the 10th edition of the European Pharmacopoeia using the sense organs. This study was carried out with three persons, and similar results obtained by the majority were accepted. Color was determined by observation with the naked eye, taste by tasting the powders, odor by sniffing and texture by touching the powders [32].

#### 4.3.2. Determination of pH

It is determined using a pH meter. After stabilization, dip the pH meter electrode in a pH 7.00 buffer solution and rinse with distilled water; place the electrode in the plant material or lyophilizate and shake until the pH is stable, take the pH reading and, finally, rinse the electrode with distilled water.

#### 4.3.3. Residual Moisture Content

The residual moisture content of the plant material or Balanites lyophilizes was determined by the gravimetric method.

A test sample of 1 g of powder in an aluminum dish was placed in a halogen desiccator at a temperature of 105 °C for 15 min. After the allotted time, the RMC value is displayed, calculated according to the following formula:

RMC = [(Mi − Mt)]/Mi × 100

Mi: test sample
Mt: mass after drying

#### 4.3.4. Extraction Yield

The extraction yield is determined by the following formula:

R = (P × 100)/P0

R: extraction yield
P: weight (g) of the dry extract obtained after lyophilization
P0: weight (g) of the plant material test sample.

### 4.4. Determination of Phytochemical Profile of Plant Material and Lyophilizate

Phytochemical screening of extracts is performed by thin-layer chromatography on chromatophores according to the methods described by Wagner and Bladt. The metabolites sought are saponins, terpene and steroid compounds, flavonoids, tannins, etc.

Thin layer chromatography (TLC) is a separation technique in which the compounds to be separated are absorbed and partitioned according to their affinity between a solid phase (silica gel) and a mobile phase (migration solvent). TLC analyses are performed according to the method described by Wagner and Baldt [51]. A 20 mg mass of lyophilizate was dissolved in 5 mL of distilled water. A 2 µL volume of the sample obtained was applied to a Chromatographic plate (Silica 60 F254; 10 cm × 5 cm; rigid aluminium support). Deposits are eluted along a 7 cm path in a glass tank containing a solvent system whose composition depends on the chemical group of interest. After elution, the Chromatographic plate are removed and dried at room temperature (25 °C), then in a ventilated oven (40 °C) for 5 min. Quercetin, a flavonoid, and diosgenin, a phytosteroid sapogenin isolated from plants, were used as standards and migrated with the extracts. The elution solvents for saponins were ethyl acetate, methanol and water in the proportions 20/3.3/2.7. Flavonoids were eluted in ethyl acetate, methanol and water in 20/3.3/2.7.

### 4.5. Saponins Contents

The following color-developing reagent solutions were prepared: (A) 0.5 mL p-anisaldehyde and 99.5 mL ethyl acetate, and (B) 50 mL concentrated sulfuric acid and 50 mL ethyl acetate. Two milliliters of 1 mg/mL diluted extract solution was added to a 10 mL test tube. After shaking, the test tube was placed in a water bath maintained at 60 °C for 10 min to develop color and then allowed to cool in a water bath maintained at room temperature for 10 min. Since the boiling point of ethyl acetate is 76 °C, the temperature of the water bath must be carefully controlled. The absorbance of the developed color solution was measured. Methanol was used as a control for the absorbance measurement. Solutions containing diosgenin and 2 mL of methanol were used to obtain a calibration curve [52].

### 4.6. Pharmacological Study

#### 4.6.1. ABTS^+^ Free Radical Scavenging Assay

The original ABTS+ assay was based on the activation of metmyoglobin with hydrogen peroxide in the presence of ABTS to produce the radical cation in the presence or absence of antioxidants. A more appropriate format for the assay is a decolorization technique in which the radical is directly generated in a stable form prior to reaction with putative antioxidants. The ABTS^-+^ radical scavenging assay was used to determine the antioxidant capacity of the extract according to Re et al. [53]. ABTS cation radical was generated by mixing in water to a concentration of 7 mM. ABTS radical cation (ABTS^-+^) was generated by reacting ABTS stock solution with 2.45 mM potassium persulfate and allowing the mixture to stand in the dark at room temperature for 12–16 h before use. Ethanol was added to the prepared solution to adjust the absorbance. To evaluate the extract, 200 µL of ABTS^-+^ radical solution was added to 20 µL of extract in a 96-well microplate. The blank for this assay was a mixture of ABTS and ethanol. Absorbance was measured at 734 nm using a BioRAd spectrophotometer (BioRad model 680, Tokyo, Japan) after 30 min incubation in the dark at 25 °C. Trolox was used as a positive control. The data were the mean of three determinations. The antioxidant capacity using the ABTS method was expressed as trolox equivalent antioxidant capacity (TEAC).

#### 4.6.2. Ferric-Reducing Antioxidant Power (FRAP) Assay

The reducing power of the extracts was determined by a method previously described by Ibe et al. [54]. A concentration of 1 mg/mL (0.5 mL) of sample extracts was mixed with 1.25 mL (0.2 M, pH 6.6) sodium phosphate buffer and 1.25 mL 1% potassium ferricyanide. The mixture was vortexed and incubated at 50 °C for 30 min. After incubation, 1.25 mL of 10% trichloroacetic acid (TCA) (*w*/*v*) was added, and the mixture was centrifuged at 1000 rpm in a refrigerated centrifuge for 10 min at room temperature. The top layer (0.625 mL) was mixed with 0.625 mL of deionized water and 0.125 mL of 0.1% ferric chloride (FeCl_3_), and the absorbance of the reaction mixture was evaluated at λ max of 700 nm against the corresponding extract concentration, and ascorbic acid was used as a positive control. The assay was performed in triplicate.

### 4.7. In Vitro Evaluation of Larvicidal Activity Against Heligmosomoides Bakeri

The tests were conducted with the extracts that gave the best yields and maintained their physicochemical characteristics. These were the 5% and 10% extracts of *B. aegyptiaca* seeds. To test the effects of the extracts, 1 mL of a solution containing 50–60 parasite larvae was added to 14 Petri dishes (10 mm by 35 mm diameter) at two concentrations [55]. The concentrations were 0.39, 1.56, 3.125, 6.25, 12.5 and 25 μg/mL for the different extracts. The tests were repeated 6 times for each treatment and the controls. Levamisole (Lev) was used as a positive control at concentrations of 1, 6.25, 12.5, 25, 37.5 and 50 μg/mL. The plates were covered, and the larvae were incubated at room temperature for 24 h, after which 2 to 3 drops of formalin were added to each plate to fix the different stages of the parasite life cycle. At the end of the time, the number of dead or immobile larvae per Petri dish was counted using a light microscope (at 10× magnification). The adjusted mortality rate (%) is calculated according to the method of Wabo et al. [56]:

$$Mc\%=\frac{Mce-Ms}{100-Ms}\times 100$$

with: Mc % corrected mortality rate
Mce is the mortality rate during the test
Ms is the mortality rate obtained with the control negative

### 4.8. Statistical Analysis

The mortality rates were analyzed with GraphPad Prism 10.0.2 software. Two-way ANOVA followed by the “Tukey” multiple comparison test was used to compare the extracts. *p* < 0.05 was considered significantly different. The results were expressed as mean ± standard error of the mean (SEM). The variation was considered significant at *p* < 0.05. *ns* is considered not significant. *n* = 3. Statistical significance was defined as * *p* < 0.05, ** *p* < 0.01, *** *p* < 0.001 and **** *p* < 0.0001.

## 5. Conclusions

This study aimed to determine the optimal conditions for the extraction of the seeds of *B. aegyptiaca* Del. (Zygophyllaceae), used in formulating a phytomedicine in Burkina Faso by varying two parameters (mass/volume ratio and time) and evaluating the extraction yield, saponins content in lyophilizates, RMC and pH. Quality control and antioxidant activity were also evaluated.

A mass/volume ratio of 10% for 12 h of maceration is the optimum parameter. The lyophilized extract obtained under the 10% to 12 h conditions showed the highest yield with the highest saponins content. RMC, pH and quality control were in accordance with plant raw material quality and preservation standards. Antioxidant activities showed no significant differences among the different extracts.

In view of these results, aqueous maceration with the parameters of 12 h maceration with a mass/volume ratio of 10% can be considered as the optimal operating conditions for the extraction of *B. aegyptiaca* seeds for the formulation of phytomedicine. This is the first established standard for *B. aegyptiaca*. These results support the use of the plant in the treatment of human and veterinary intestinal parasitosis.

## Figures and Tables

**Table 1:** Macroscopic and organoleptic characteristics of *Balanites aegyptiaca* seeds powder and lyophilizate.

Characteristics	Plant Material	Lyophilized Extracts
Color	Yellow	Light yellow
Odor	Very characteristic	Characteristic
Taste	Amer	Amer-sweet
Texture	Coarse	Fine

**Table 2:** pH of lyophilizates.

Maceration Time (h)	pH				
	5%	10%	20%	30%	40%
6 h	6.99 ± 0.04	6.99 ± 0.01	6.89 ± 0.01	6.81 ± 0.01	6.81 ± 0.02
12 h	6.82 ± 0.01	6.87 ± 0.02	6.88 ± 0.01	6.85 ± 0.01	6.83 ± 0.02
24 h	6.43 ± 0.02	6.61 ± 0.02	6.54 ± 0.01	6.60 ± 0.02	6.67 ± 0.01

**Table 3:** Extraction yield of aqueous macerates mass/volume ratio as a function of time (hours).

Maceration Time	Yield (%)				
	5%	10 %	20%	30%	40%
6 h	0.33 ± 0.009 ^ab^	0.27 ± 0.013	0.22 ± 0.01	0.22 ± 0.012 ^b^	0.22 ± 0.011
12 h	0.28 ± 0.011	0.35 ± 0.01 ^abc^	0.22 ± 0.012 ^b^	0.23 ± 0.009	0.23 ± 0.011 ^b^
24 h	0.23 ± 0.008	0.21 ± 0.01	0.20 ± 0.01	0.20 ± 0.01	0.18 ± 0.01

^a^: high content versus time parameter, ^b^: high content versus mass/volume ratio parameter and ^c^: high content versus time and mass/volume ratio parameters.

**Table 4:** Phytochemical groups detected in lyophilizates.

Phytochemical Components	Lyophilizates
Saponins	+
Flavonoids	+
Tannins	-
Triterpenes and sterols	-

With + = present and - = absent.

**Table 5:** Saponins content of lyophilizates.

Maceration Time	Saponins Content (mg/g)				
	5%	10%	20%	30%	40%
6 h	10.51 ± 0.01	11.83 ± 0.03 ^a^	11.07 ± 0.07	10.56 ± 0.02	9.97 ± 0.04
12 h	12.32 ± 0.07 ^b^	13.81 ± 0.04 ^bc^	11.61 ± 0.02 ^b^	12.29 ± 0.06 ^b^	12.39 ± 0.02 ^b^
24 h	1.34 ± 0.05	11.46 ± 0.04	9.88 ± 0.09	9.27 ± 0.07	9.11 ± 0.01

^a^ = high content concerning the time parameter. ^b^ = high content in the mass/volume ratio parameter. ^c^ = high content concerning the time and mass/volume ratio parameters.

**Table 6:** IC_50_ of extracts by ABTS method.

Maceration Time	50% Inhibitory Concentration (5 ug/mL)				
	5%	10%	20%	30%	40%
6 h	4.82 ± 0.10 ^abc^	4.54 ± 0.13 ^b^	4.07 ± 0.15 ^b^	3.93 ± 0.10	4.35 ± 0.18
12 h	4.52 ± 0.19	4.25 ± 0.12	4.02 ± 0.17	3.98 ± 0.12 ^b^	4.36 ± 0.13 ^b^
24 h	3.84 ± 0.15	4.03 ± 0.13	3.57 ± 0.21	3.60 ± 0.20	4.20 ± 0.17 ^a^

^a^ = high content versus time parameter. ^b^ = high content versus mass/volume ratio parameter. ^c^ = high content versus time and mass/volume ratio parameters.

**Table 7:** IC_50_ of extracts by FRAP method.

Maceration Time	Reduced Iron Content/µmolEA				
	5%	10%	20%	30%	40%
6 h	0.73	0.73	0.73	0.74	0.74
12 h	0.73	0.74	0.72	0.73	0.72
24 h	0.73	0.71	0.74	0.73	0.74

## Data Availability

All data generated or analyzed in this investigation are reported in this.
